# Proximity care pathways and digitalization: opportunities and concerns for medication safety management—Insights from the ProSafe study on community perspectives

**DOI:** 10.3389/fpubh.2025.1486814

**Published:** 2025-02-20

**Authors:** Francesca Moretti, Maria Angela Mazzi, Sara Montresor, Silvia Colpo, Ilaria Tocco Tussardi, Daniela Facchinello, Raffaella Robello, Luigi Ambroso, Cristina Destro, Salvatore Leone, Davide Petruzzelli, Michela Rimondini, Ugo Moretti

**Affiliations:** ^1^Department of Neuroscience, Biomedicine and Movement, University of Verona, Verona, Italy; ^2^Department of Diagnostics and Public Health, University of Verona, Verona, Italy; ^3^Roche S.p.A., Monza, Italy; ^4^Federazione delle Associazioni Emofilici ONLUS – FEDEMO, Roma, Italy; ^5^Women Against Lung Cancer in Europe – WALCE, Torino, Italy; ^6^Associazione Nazionale per le Malattie Infiammatorie Croniche dell'Intestino (Colite Ulcerosa e Malattia di Crohn) – A.M.I.C.I. ETS, Milano, Italy; ^7^La Lampada di Aladino ETS, Brugherio, Italy

**Keywords:** proximity care, medication safety, community engagement, community perspective, digitalization of care, health service planning, participative research

## Abstract

**Background:**

Establishing proximity care pathways, including the digitalization of healthcare, is valuable for sustainable management of Non-Communicable Diseases (NCDs) and Patient-Centered Care (PCC) promotion. However, new safety concerns, particularly in therapy management, may arise. The Community-Based Participatory Research (CBPR) “ProSafe” aims at (i) explore stakeholders’ perspectives on medication safety management in proximity care and (ii) analyze which determinants affect the community’s perspective.

**Methods:**

A survey was co-developed with a Patient Safety Council (PSC) and the support of a pharmaceutical company. A purposeful sampling strategy was implemented to recruit individuals aged 18 and older. Data were collected using a dedicated online platform; differences between patients’ and healthy people’s perspectives were explored. Preliminary multiple regression analyses were performed to examine how sociodemographic factors, clinical data and level of digitalization affect outcomes using linear and probit models, accounting for the nature of each outcome variable. The models were combined into multiple equations using a Conditional Mixed Process (CMP) approach.

**Results:**

417 individuals completed the survey (81.0% affected by a disease). A positive attitude towards shifting therapy administration from hospital to home setting was observed even if a significantly higher proportion of patients compared to healthy individuals raised concerns regarding a potential negative impact on the doctor-patient relationship (47.0% vs. 32.9%, *p* < 0.01). Additionally, 63.7% of patients reported they would feel less supported in the care process. The usefulness of telehealth, including tele-pharmacy for drug therapy management, was rated higher by healthy individuals compared to patients (mean value 1.3 vs. 1.5 *p* < 0.01); 43.9% of patients raised concerns regarding the excessive responsibility placed on them in digital care compared to traditional healthcare. Health status and level of education were the variables most frequently associated with significant impacts across multiple outcomes.

**Conclusion:**

The community’s perspective on the development of proximity care pathways provided valuable insights into concerns, fears, and limitations that could impact the effectiveness of this important shift in healthcare delivery. Effectively addressing these issues is essential to truly bring disease and medication management closer to patients and their living environments while ensuring that the community becomes co-creators in the implementation of proximity care, fostering health equity and patient autonomy.

## 1 Introduction

The spread of Non-Communicable Diseases (NCDs), together with population aging, represents a challenge for healthcare systems requiring the development of new organizational models capable of addressing patients’ care needs in an equitable, personalized, and efficient way (1). Globally, the prevalence of NCDs continues to grow, driven not only by unhealthy lifestyles but also by insufficient healthcare service provision (2, 3). In this context, the COVID-19 pandemic has further highlighted the vulnerabilities within healthcare systems worldwide, emphasizing the urgent need to prioritize sustainable development Initiatives such as NextGenerationEU aim to enhance resilience and foster innovation across sectors, with a particular focus on transforming healthcare to meet these pressing challenges (4, 5).

In Italy, the response to these imperatives is exemplified by Mission Health 6, a pivotal component of the National Recovery and Resilience Plan (NRRP), which introduces a vital reform measure to reorganize and strengthen territorial healthcare (6). While healthcare policies are set nationally, in Italy, regional authorities play a crucial role in tailoring services to the specific needs of the local population. Territorial healthcare is organized through Local Health Authorities, who are responsible for identifying the collective health needs of their resident population, planning healthcare assistance and pathways, and providing all necessary services (7).

Key elements of the NRRP’s vision are the creation of integrated multidisciplinary proximity pathways (including the promotion of home care solutions), the establishment of new intermediate care facilities situated between intensive hospital care and low-intensity home care, such as community hospitals, and the digitalization of healthcare (including telemedicine). The goal is to bring prevention, medication treatment, and rehabilitation closer to the community’s living environment, enabling more personalized and responsive care, particularly for managing NCDs (6). Several other countries are also investing in a comparable territorial reorganization, and the digitalization of care represents a global challenge (8, 9).

Beyond the inherent potential of these organizational solutions, safety concerns, including new challenges in therapy management, may arise. Medication errors are a crucial priority for patient safety, and the complexities of the home care setting expose patients to a high risk for medication mismanagement, with evidence suggesting that over 40% of home care recipients may be affected by these issues (10). Furthermore, the opportunity to move therapy administration closer to patients’ living environments, shifting the setting of medications generally administered in the hospital to a community-based setting, highlight the importance of proactively identifying potential safety concerns to ensure the safe management of medical products (11, 12). Additionally, implementing various digital health tools and technologies to facilitate communication and information sharing regarding drug management among healthcare providers (HCPs), patients, and caregivers may introduce new safety issues that must be promptly identified and adequately addressed (13, 14).

To fully explore and adequately handle novel safety challenges, the perspectives of key stakeholders in the PNRR reform, including healthy citizens, patients, caregivers, and HCPs, are valuable. As underlined by the Institute for Patient- and Family-Centered Care, Person-Centered Care (PCC) can be defined as “*planning, delivery, and evaluation of health care that is grounded in mutually beneficial partnerships among healthcare providers, patients, and families*” underlying how community engagement is warranted at all levels of healthcare provision including health services development and delivery care organization (15). Evidence shows how such a community engagement in health services planning and implementation leads to positive results at various levels, including improved service utilization (e.g., decrease in avoidable hospitalizations), increased community knowledge and awareness, and safety improvement, such as enhanced drug management (16). According to a recent Cochrane systematic review, other significant results included improved health service design and delivery, such as the opportunity to provide “*medical treatment closer to home*” (17).

A Community-Based Participatory Research (CBPR) project named “ProSafe” has been developed within this context. CBPR is a research approach that actively involves community members at every stage of the research process, ensuring their valuable participation and contribution (18, 19). This approach guarantees that interventions are not only scientifically valid but also culturally relevant, practical, and aligned with the needs and priorities of the community (20). The “ProSafe” project engages academic researchers, an advisory board including members of the boards of directors of four national patient associations (PAs), collectively referred to as the Patient Safety Council (PSC), and a pharmaceutical company.

PAs within the PSC represent individuals living with or caring for conditions that place a substantial burden on healthcare resources and have a significant impact on quality of life. These conditions include chronic inflammatory bowel disease (AMICI), congenital coagulation disorders (FedEmo - Federation of Hemophilia Associations), and cancer (WALCE - Women Against Lung Cancer in Europe and ‘La Lampada di Aladino’). The “ProSafe” project seeks to gather insights from citizens, patients, and HCPs across various disciplines and roles, on critical issues related to the development and implementation of proximity care and the integration of digital care, emphasizing emerging medication safety concerns. A recent systematic review of the literature focused on identifying patient safety risks associated with the use of telecare in home care services, identified 11 types of safety risks, including organizational issues and technological or device-related challenges, highlighting the importance of involving the community in developing concrete solutions to mitigate these issues (21). To our knowledge, no studies have yet investigated this fundamental concern. Specifically, the primary aims of the ProSafe project are: (i) to explore stakeholders’ perspectives on the opportunities and potential new safety challenges arising from the reorganization of proximity medicine and the shift of therapies closer to patients’ living environments, including the promotion of digitalization; (ii) to analyze how socio-demographic, clinical, and personal factors, such as the desired participation in medication choice and the level of digitalization, impact these perceptions; (iii) to compare viewpoints between the community and HCPs, and (iv) to identify and implement actions to improve medication safety in this emerging proximity care context.

This publication addresses goals 1 and 2 of the ProSafe study, while goals 3 and 4 will be discussed in future publications.

## 2 Materials and methods

### 2.1 Study design

The ProSafe project is a CBPR study developed through an active partnership between the university and community members. Specifically, the PSC was an integral and essential part of the research team, actively engaged in all project stages, including defining the aims and methods, data collection and analysis, data synthesis, results reporting, and disseminating the key findings.

The project implemented a cross-sectional design, and a survey (named “ProSafe community survey”) was co-developed with the PSC. The pharmaceutical company Roche supported the partnership between the academic partners and the PSC throughout the project’s development.

The project was approved by the Local Ethics Committee. A detailed description of the PSC’s engagement and project methodology is provided elsewhere (22).

### 2.2 Study population and recruitment method

Based on the multi-point snowball technique, a purposeful sample strategy was implemented to recruit participants aged 18 years or older, with no other exclusion criteria, who were interested in providing their perspectives (23). Participation was entirely voluntary, and no incentives were provided to participants. Given the complexity of the topic and the aim of gathering as much information as possible, it was presumed that individuals involved in PAs would have a more specific understanding. Therefore, the recruitment process was initiated through the following channels: (1) Memberships of the PAs affiliated with the PSC were reached via social media (Facebook, Twitter, Instagram), association websites, flyers at association offices, and a dedicated newsletter sent to all members; (2) Other PAs within the pharmaceutical company’s network were reached via a dedicated email. Participants were then invited to share the link within their networks.

### 2.3 Instrument: the ProSafe community survey

#### 2.3.1 Instrument co-creation process and stakeholder contributions

In alignment with the core principles of a CBPR project, academic researchers and the PSC worked in partnership to co-create the questionnaire, identifying key themes and refining the instrument in an iterative process (24).

The PSC played a crucial role in determining the main areas of focus for the survey, ensuring that the insights generated would be relevant and meaningful to the target population, particularly chronically ill patients.

The primary interest of the PSC was to explore how the strengthening of home care, the enhancement of intermediate care networks, and the transition toward digitalization (as driven by reforms under the NRRP) could influence medication safety.

Specifically, the PSC identified three key themes to be addressed in the survey:

- Knowledge and perceived usefulness of organizational care solutions bridging the gap between home-based care and hospital services (i.e., intermediate healthcare facilities).
- Perceived benefits and challenges related to medication safety arising from the transition of medication administration from hospitals to community-based settings.
- Perceived benefits and challenges of digital medicine, with a focus on the usefulness of digital medication dossiers, telehealth services, and digital monitoring tools.

Moreover, as outlined in Section 3 of the paper, academic researchers collaborated with the PSC to identify a set of independent variables that could potentially influence the results and needed to be explored to ensure rigor in the findings. These included socio-demographic data, clinical data, experiences with the digitalization of care, and desired involvement in care and medication management.

After mapping out the critical issues to be addressed in the survey, a first draft of the items was developed based on the literature and national policies, and integrating feedback from the PSC to ensure alignment with community perspectives. A pre-test, consisting of cognitive interviews with a sample of 25 community members, was then conducted to integrate their perspectives, validate the survey, and refine it into its final version. Finally, a preliminary pilot study was also performed to evaluate the feasibility and data quality.

The survey development was guided by a broad definition of medication harm, described as “any negative patient outcomes or injury, related to medication use, irrespective of severity or preventability” (25). This comprehensive definition provided a shared framework for addressing medication safety and ensuring that the questionnaire captured a wide range of potential issues and outcomes.

The level of PSC involvement and contributions at each decision point in project development is detailed elsewhere (22). However, in line with CBPR principles, decision-making power was equitably distributed between researchers and co-researchers across all stages of the project, including planning, data collection, data synthesis, results dissemination, and action planning.

Regarding the role of the pharmaceutical company, it initially established the PSC with the primary aim of raising awareness about patient safety in pharmacological treatments. For the ProSafe project specifically, the company facilitated the initial connection between academic partners and the PSC, as well as supported the organization of meetings that were tailored to the project’s needs, taking into account the specific contributions of both parties.

#### 2.3.2 Survey items and overall structure

The final version consisted of 30 questions distributed across five sections.

Specifically, Section 1 included 15 questions to collect sociodemographic and clinical data.

Section 2 consisted of one question examining the desired level of engagement in medication choice and three questions assessing the level of digitalization. These included knowledge on how to access the Electronic Health Record (HER), habitual use of digital platforms (from a choice of 5 options), and accessibility to all necessary equipment for telemedicine (offering four different options).

Section 3 comprised eight questions across two different areas. Area 1 focused on knowledge, perceived needs, and attitudes toward the reorganization of proximity care, specifically emphasizing proximity medication management and related safety issues; Area 2 addressed the digital evolution in proximity medicine development and its potential impact on medication safety.

Section 4 explored the utilization of digital drug support tools (1 question) and participants’ perceptions of helpful content for digital support tools in medication safety (1 question with options rated on a 3-point Likert scale). Finally, Section 5 included an open-ended question inviting participants to express any concerns or issues regarding medication safety that they would like to address further.

The last two sections will be analyzed in a subsequent publication.

### 2.4 Explanatory and outcome variables

Sections 1 and 2 encompass all the explanatory variables considered for the study.

Section 3 comprises the eight outcome variables (4 for Area1 “Knowledge, perceived needs, and attitudes toward the reorganization of proximity care and medication safety issues” and 4 for Area2 “Digital Evolution in proximity care development and impact on medication safety”).

Information was synthesized by calculating means for variables measured on a Likert scale or as count variables (e.g., indicating the number of options selected out of a maximum, such as “Which of the following digital applications do you normally use?”). Scores for negatively formulated items on Likert scales were reversed as necessary.

### 2.5 Data collection

The data collection utilized a dedicated online platform developed by the University of Verona. An access link to this platform was generated for distribution across social media channels to reach all potential participants. Through this platform, participants could access and complete the online survey from the 1^st^ of May to the end of October 2023.

### 2.6 Sample size calculations and data analysis

Regarding the sample size, given that the community survey was distributed nationally to the entire population with no exclusion criteria (except age), the population size can be around 50 million (ISTAT, 2023). A sample of 400 subjects is required, according to Lynch’s formula, which calculates a minimum size of 385 subjects for the following specified criteria: confidence level at 95%, margin of error of 5%, and response distribution fixed to the most conservative assumption (50% for the proportions) (26).

Descriptive statistics were used to report the main findings, while Student’s t and chi2 tests were applied, where appropriate, to explore the main differences between the groups of patients and healthy people.

Regarding the second aim of this study, multivariate explorations were performed in steps to have two final models, one for each of the two areas of investigation, following a parsimonious criterium to identify the explanatory variables. Within a Generalized Linear Model (GLM) framework, the identity and probit transformations (link) of the dependent variable were chosen, considering the nature of the outcome variable: the linear fit of the continuous outcomes, despite their limited interval range due to average values expressed on Likert scales, was supported by recent literature exploring potential biases and information loss when using Likert scales (27). Regarding dichotomous outcomes, the probit was preferred over the logit link because it only assumes the constraint of the normal distribution of the residuals (28).

Below are the details of the two steps taken in the analysis:

1. For each of the 8 outcomes distinctly, a preliminary selection procedure of the potential predictors was performed, based on hierarchical “block-wise” regressions. At first, a multiple regression model was estimated for the demographic block; the variables showing a relevant effect (*p*-value <0.10) on outcome were included in the following regression. This procedure was repeated for the block of the clinical characteristics and then for digitalization attitudes. The eight sets of selected explanatory variables were used as a starting base for the following step.
2. For each target area, a multivariate model comprehensive of the four outcomes was estimated by applying the Conditional Mixed Process (CMP) technique. This simultaneous estimation approach is specifically useful to solve a system of independent equations with correlated residuals and to identify more efficient estimates than those derived from separate regression models. It can be considered an extension of the “seemingly unrelated regression” (SUR), which is constrained to linear regression equations, focused to include GLMs too. In order to check the assumptions of the CMP a residual diagnostic analysis was performed as reported in the Supplementary material (Supplementary Figures S2, S3; Supplementary Tables S5, S6).

Stata 18 was used to perform the analyses (29); more specifically, the package “cmp,” based on the maximum likelihood estimation and specifically built to estimate the fully observed recursive mixed-process modes, was adopted to jointly handle continuous and binary outcome variables (30).

## 3 Results

### 3.1 Population characteristics: socio-demographic and clinical data

A total of 584 individuals consented to participate in the survey, and 417 (71%) returned it completed, with 337 (81.0%) declaring being affected by a disease and 79 (19.0%) declaring being healthy. Among these 79 individuals, 26 (32.9%) affirmed being caregivers. The mean age was 52 years old (range 19–86). Socio-demographic characteristics of respondents among the total and stratified by the presence/absence of disease are reported in Table 1.

**Table 1:** Socio-demographic characteristics of the sample.

	Total (*N* = 417)	Patients (*N* = 337)	Healthy individuals (*N* = 79)	Chi2 (*p*)
Gender	N (%)	%	%	1.37 (0.24)
Female	243 (64%)	62.5%	70.0%	
Male	137 (36%)	37.5%	30.0%	
Age class				
<30	29 (7%)	6.3%	10.1%	**11.95 (<0.01)**
30–49	130 (31%)	28.4%	44.3%	
50–69	215 (52%)	54.3%	41.8%	
≥70	40 (10%)	11.0%	3.8%	
Academic degree				0.92 (0.63)
ES^a^/JHS^b^ Diploma	36 (9%)	9.2%	6.3%	
High School Diploma	205 (49%)	49.6%	48.1%	
University/ PG^c^ Degree	175 (42%)	41.2%	45.6%	
**Origin ^**				**16.99 (<0.01)**
North Italy	270 (65%)	60.2%	84.8%	
Center Italy	41 (10%)	11.3%	3.8%	
South Italy	105 (25%)	28.5%	11.4%	
Health status self-perception				**49.83 (<0.01)**
Excellent	33 (8%)	3.9%	25.3%	
Good	208 (50%)	49.0%	55.7%	
Fair	125 (30%)	33.7%	15.2%	
Poor	48 (12%)	13.4%	3.8%	
Member of a patients’ association (% yes)	260 (64%)	71.1%	33.3%	**39.03 (< 0.01)**

*Percentages are calculated based on the obtained answers excluding missing data; ES, elementary school; ^b^JHS, junior high school; ^c^PG, postgraduate; ^According to ISTAT classification. Significant differences are highlighted in bold.

Table 2 shows the main clinical characteristics of the subsample affected by a disease.

**Table 2:** Clinical data of patients’ subsample (*N* = 337).

	*N* (%)*
Main pathology	
Non-oncological	208 (66.7)
Oncological	82 (26.3)
Hereditary/congenital	22 (7.0)
Time since diagnosis	
< 1 year	18 (5.7)
1–5 years	102 (32.5)
6–10 years	49 (15.6)
>10 years	145 (46.2)
Comorbidity	
Yes	145 (45.0)
No	177 (55.0)
Number of medications/day	
None	12 (3.7)
1 medication	81 (24.9)
2–5 medications	185 (56.7)
>5 medications	48 (14.7)
Regular hospital check-ups for medication	
Yes	133 (45.5)
No	159 (54.5)
Perceived support	
Insufficient	32 (10.3)
Sufficient	87 (27.9)
Good	119 (38.1)
Excellent	74 (23.7)

*Percentages are calculated based on the obtained answers, excluding missing data.

Among individuals who stated that they needed to undergo regular hospital check-ups because of their medications, 25% reported experiencing side effects.

### 3.2 Explanatory variables: desired participation in medication choice and level of digitalization

Sample characteristics regarding explanatory variables from Section 2 of the survey are reported in Table 3.

**Table 3:** Explanatory and outcome variables for the entire sample.

Variable	Items		Measure		
	*N*	Type scale	Range	Mean or %	IC95%
Section 2					
Desired participation in medication choice	3	Likert 1-4	1-4	3.1	3.1-3.2
Level of digitalization					
Digital platforms habitually used*	5	Count	0-5	3.9	3.8-4.0
know how to access their EHR (% yes)	1	Dichotomous	0-1	72.4%	67.9-76.7
Having all the necessary equipment for a telemedicine visit**	4	Count	0-4	3.8	3.7-3.9
Section 3					
Area 1. Knowledge, perceived needs, and attitudes toward the reorganization of proximity care and medication safety issues					
Knowledge of IF (% yes)	1	Dichotomous	0-1	23.1%	19.1-27.4
issues with continuity of care from hospital to Home/Community (% yes, at least one issue)	3	Dichotomous	0-1	70.1%	65.5-74.5
Attitudes toward shifting hospital therapy to the home-setting	8	Likert 1-4	1-4	2.8	2.8-2.9
Attitudes toward shifting hospital therapy to the IF setting	7	Likert 1-4	1-4	2.8	2.7-2.8
Area 2. Digital evolution in proximity care development and impact on medication safety					
The propensity toward the implementation of the digital pharmaceutical dossier (% completely favorable^§^)	2	Dichotomous	0-1	40.0%	35.3-44.9
Perceived usefulness of telehealth visits/consultations for monitoring therapy	3	Likert 0-2	0-2	1.3	1.3-1.4
The propensity toward digital medicine (vs. traditional medicine)	9	Likert 1-4	1-4	3.0	2.9-3.0
The propensity toward digital monitoring (vs. in-person monitoring) - % of “better and more personalized	1	Dichotomous	0-1	26.2%	22.0-30.7

IF, intermediate facilities; *Among 5 choices: emails, instant messaging services, social media, search engines, and platforms for teleconferencing or video calls (from 0 to 5); ** Having an internet connection, a PC smartphone or tablet with a photo camera, and an application for video calls; ^§^Percentage calculated as the proportion of people who completely agree with positive items and completely disagree with negative items.

Concerning desired participation in medication choice, 96.1% of the sample agreed that “*It’s important to be informed about all the possible side effects of medications*,” 93.7% agreed that “*Being involved in decisions about medications increases confidence and reduces the likelihood of interruption*,” and 82.1% agreed that “*It is entirely the doctor’s responsibility to choose the best medication option*.”

No difference between patients and healthy individuals was detected regarding the level of digitalization (Supplementary Tables S1, S2). Results per item showed that 89.4% of participants have the application for video calls for a telehealth consultation, while 59.5% reported regularly using it in everyday life.

### 3.3 Outcome variables

The main results for Area 1 and Area 2 for the entire sample are reported in Section 3 of Table 3. The distribution of answers by item with comparisons between subsamples of patients and healthy individuals is detailed in Supplementary Table S2.

Among the explored outcomes, healthy subpopulations reported significantly higher scores for the outcomes of Area 1, *“Attitudes toward shifting hospital therapy to home-setting,”* and *“Attitudes toward shifting hospital therapy to IF-setting*,” and outcomes of Area 2, “*Perceived usefulness of telehealth visits/consultations for monitoring therapy,”* and “*Propensity towards digital medicine (vs. traditional medicine)*” (see Supplementary Table S1).

Among Area 1, regarding continuity of care from hospital to home/community setting, 50.6% of the sample reported a “*sudden worsening of a chronic disease difficult to manage at home but not serious enough to warrant hospitalization*” (56.1% of patients vs. 27.8% of healthy individuals, *p* < 0.01); 42.5% experienced the “*feeling of being discharged from the hospital too soon*”; and 31.6% encountered “*difficulty in continuing pharmacological treatment at home after a hospital discharge,*” such as problems in obtaining newly prescribed medications (52.3%), inadequate information regarding potential adverse effects and interactions with other treatments (38.6%), or medication reconciliation failure (26.5%). Additionally, 2.2% of the sample reported being readmitted to the hospital.

**Figure 1:**
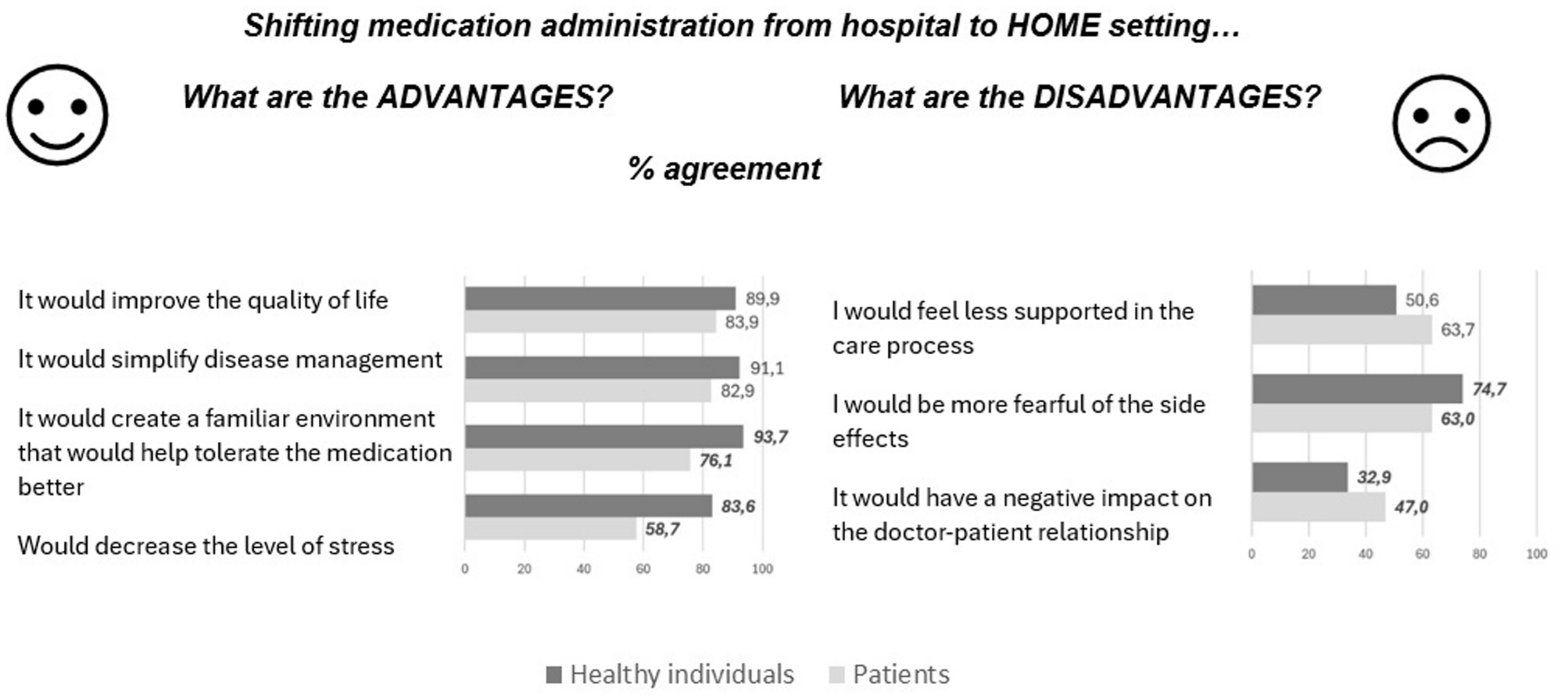
Distributions of answers (% of agreement) regarding advantages and disadvantages of shifting medication administration from hospital to home setting by subpopulations of patients and healthy individuals. Significant differences between patients and healthy individuals are highlighted in bold.

Concerning attitudes toward shifting the administration of medications typically given in the hospital setting to a community-based setting, 85.5% of the sample agreed that “*it is, in general, a useful change*” for shifting toward a home-based setting (84.1% for patients vs. 91.1% for healthy individuals, *p* = 0.01), and 77.3% for shifting toward intermediate care setting (74.9% for patients vs. 87.2% for healthy individuals, *p* = 0.02). Figure 1 displays the distribution of responses within the patients’ and healthy individuals’ subsamples regarding the shift toward a home-based setting.

Additional details on the results of the shift toward an intermediate care setting are provided in Supplementary Figure S1.

Regarding Area 2, the digital pharmaceutical dossier as a specific part of the EHR was considered a “*valuable resource to identify all potentially dangerous interactions or incompatibilities between medications*” by 95.2% of the sample. Additionally, 22.2% of responders agreed it is “*an initiative that risks reducing patient involvement in therapy management*” (item 2, negative).

The “*usefulness of telehealth visits and consultations for drug therapy management*” was rated as “*essential*” by a significantly lower proportion of patients compared to healthy individuals, with percentages, respectively, of 36.0% vs. 56.2% (*p* < 0.01) for teleconsultations with the general practitioner (GP) or specialist (telemedicine), 32.0% vs. 49.4% (p < 0.01) for telehealth visit with community nurses or other HCPs, and 33.8% vs. 45.6% (*p* = 0.05) for tele-pharmacy services. The proportion of “*not useful*” answers was 5.3, 7.3, and 7.5%, respectively.

**Figure 2:**
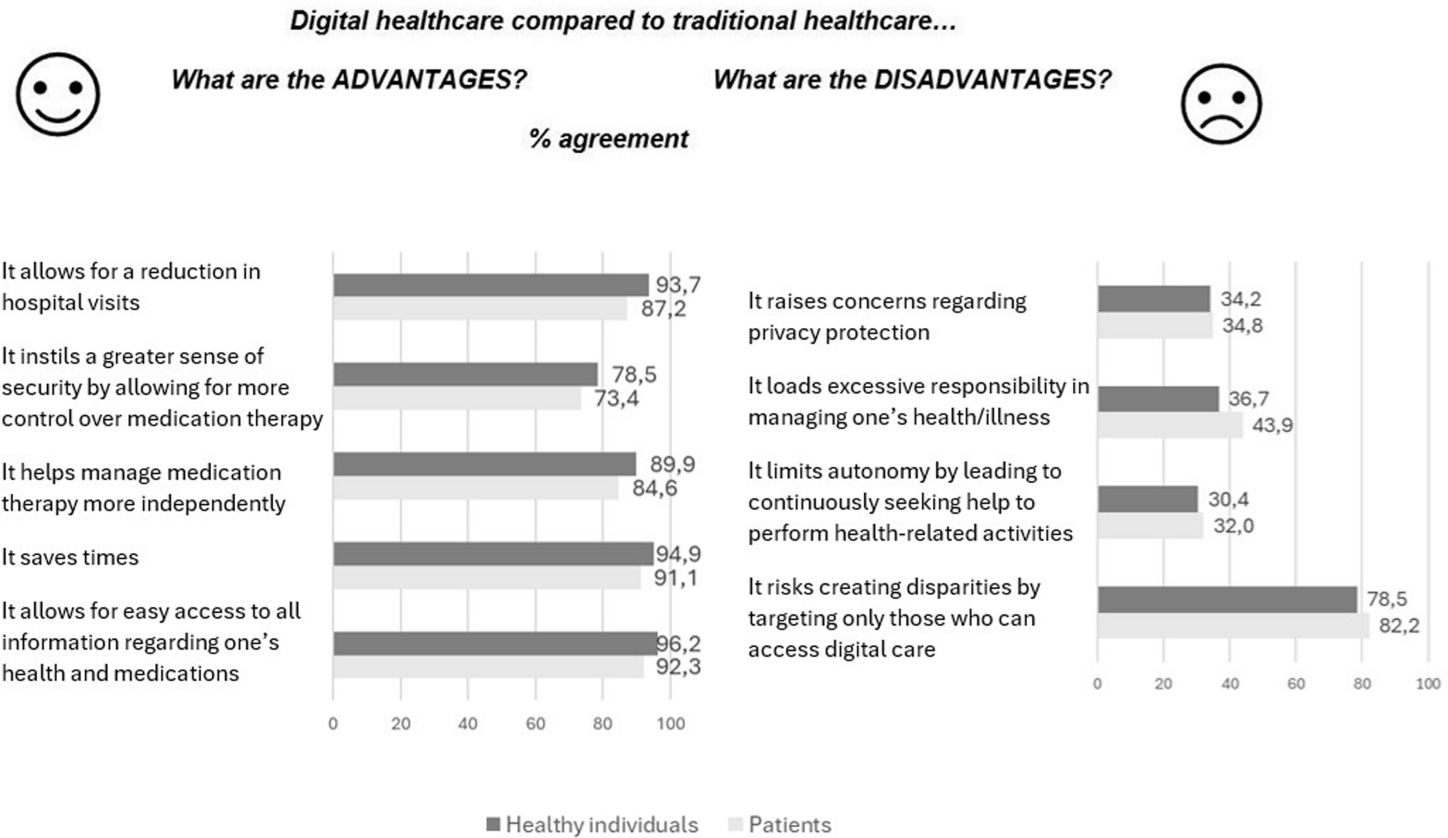
Distributions of answers (% of agreement) regarding advantages and disadvantages of digital healthcare compared to traditional healthcare, stratified by subpopulations of patients and healthy individuals.

Regarding preferences toward digital medicine versus traditional medicine, significantly lower scores were observed in the patient subsample (Supplementary Table S1). Figure 2 details the distribution of answers stratified by items among patient and healthy individual subsamples.

**Figure 3:**
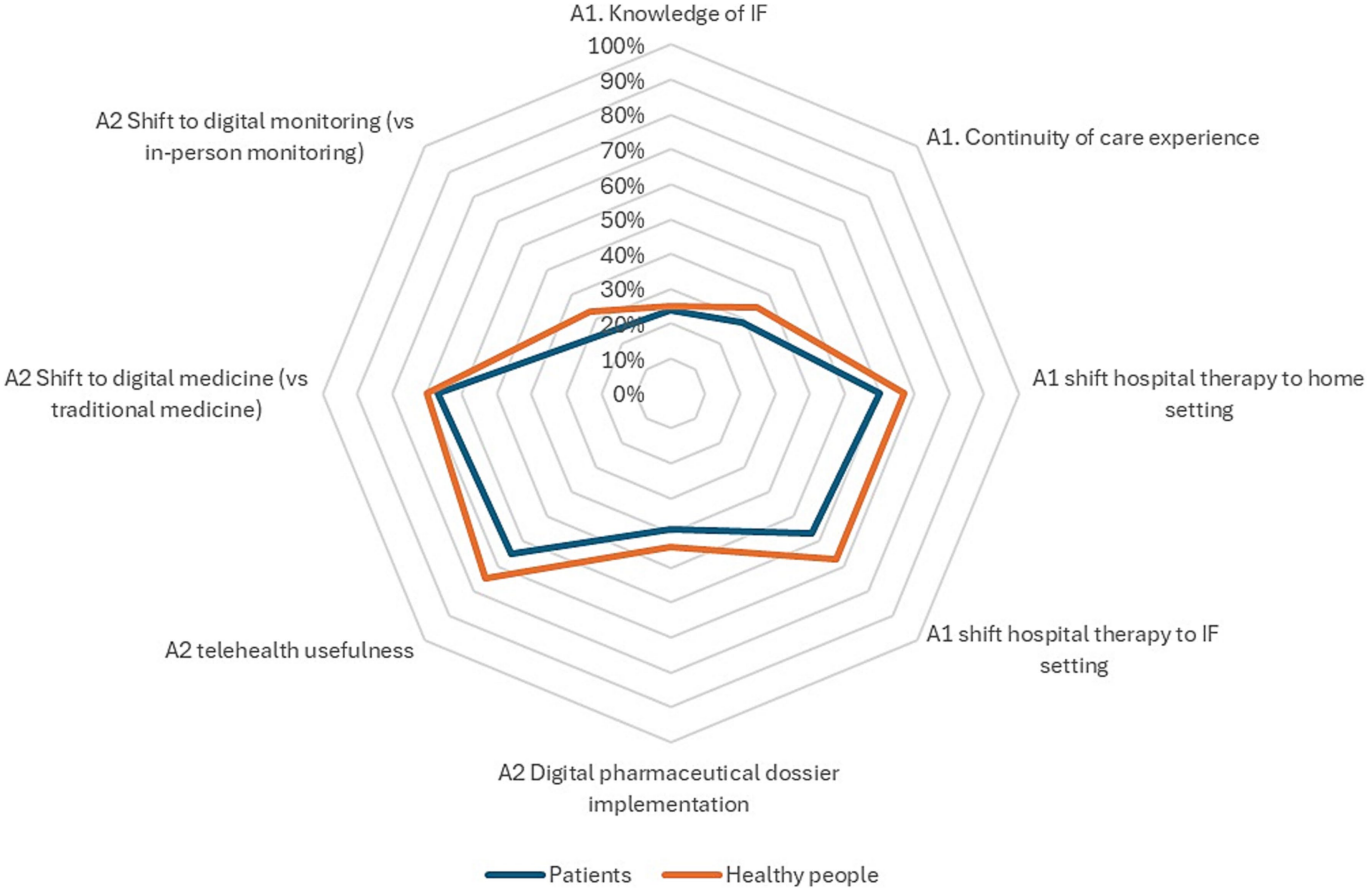
Graphic representation of the main ProSafe results. Each point on the target figure represents the outcome for one of the 8 measures, with results on a 100-point scale. Scores for negative outcomes (e.g., issues with continuity of care) were reversed for comparison. The boundaries of the target figure indicate a completely positive result, with a larger area reflecting more positive outcomes.

Figure 3 summarizes the main results among the eight explored outcomes. Scores for each outcome are presented on a 100-point scale, allowing for comparison. Outcomes closer to the edges (100% score) indicate more positive results or greater alignment with a thoroughly positive attitude. The patient’s area is narrower than healthy individuals despite a similar pattern observed between the two subpopulations.

### 3.4 Conditional mixed-process results

**Figure 4:**
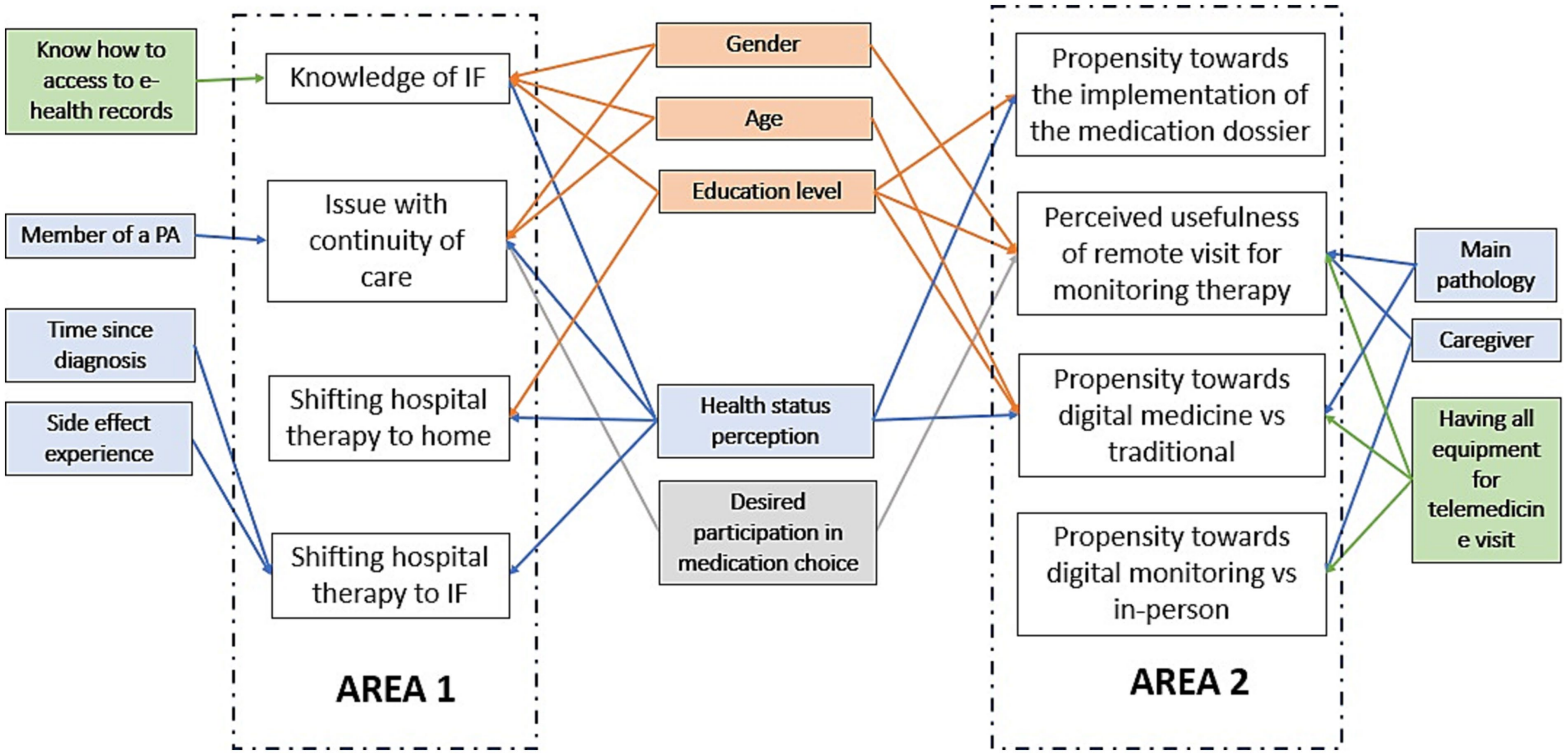
Path diagram representing the associations among the 8 outcomes and the participant characteristics, moving from the two estimated CMP models of Area A and B. White rectangles represent the 4 outcomes of each model, respectively named Area A and B, and limited by the dotted lines. The other rectangles are the participant characteristics, split by blocks: demographic (orange), clinical (blue), digitalization (green), and desired engagement (grey). Arrows represent the relevant associations between explanatory variables and dependent variables. The estimates are detailed in the Supplementary Tables S3, S4.

Figure 4 illustrates the regression paths linking significant independent variables to each of the eight outcomes across the two areas investigated in the ProSafe study.

The variable health status perception was shown to impact six different outcomes significantly. Specifically, higher perceived levels of health were associated with fewer issues with continuity of care from hospital to home/community setting (Coeff = −0.35, CI95%:-0.55;-0.16), a higher proportion of people aware of IF (Coeff = 0.2, CI95%:0.13-0.41), more positive attitudes toward shifting medication administration from hospital to home setting (Coeff = 0.10, CI95%:0.03-0.17) or intermediate care setting (Coeff = 0.14, CI95%:0.05-0.23), and a higher propensity toward digital medicine (compared to traditional medicine) (Coeff = 0.07, CI95%:0.01-0.12) and toward the establishment of the digital pharmaceutical dossier (Coeff = 0.17, CI95%:0.02-0.33).

Education levels were significantly associated with five different outcomes. Compared to people with a University degree or higher education, having lower levels of education was associated with a lower proportion of people aware of IF (Coeff = −7.2, CI95%:-1.38;-0.55), less positive attitudes toward shifting medication administration from hospital to home setting (Coeff = −0.21, CI95%:-0.27;-0.04), lower perceived usefulness of telehealth and tele-pharmacy (Coeff = −0.08, CI95%:-0.27;-0.11), and a lower propensity toward digital medicine (compared to traditional medicine) (Coeff = −0.02, CI95%:-0.19;0.16) and the establishment of the pharmaceutical dossier (Coeff = −0.34, CI95%:-0.80;-0.12).

Compared to healthy individuals, patients affected by oncological (Coeff = −0.21, CI95%:-0.35;-0.06) or non-oncological diseases (Coeff = −0.13, CI95%:-0.25;-0.01) showed lower perceived usefulness of telehealth and tele-pharmacy (mean values, respectively, of 1.46 vs. 1.31 vs. 1.16). The propensity toward digital medicine was significantly lower for patients affected by an oncological disease compared to healthy individuals (Coeff = −0.14, CI95%: −0.28;-0.00, mean values, respectively, of 2.80 vs. 3.10), while no significant differences were observed between healthy individuals and those with non-oncological diseases.

Finally, having all the necessary equipment for a telemedicine visit was significantly associated with a higher perceived usefulness of telehealth and tele-pharmacy consultations (Coeff = 0.14, CI95%:0.06;-0.22) and a greater propensity toward digital medicine and digital monitoring.

Regression coefficients for all significant associations retrieved from the two CMP models are reported in Supplementary Tables S3, S4.

## 4 Discussion

As confirmed by literature, the progression toward healthcare delivery that is increasingly ‘closer’ to patients’ needs and their living environment is a desirable goal globally (31). In this regard, the findings of the ProSafe study provide a valuable source of information and reflection. Indeed, the ProSafe project has brought to light patient- and citizen-centered perspectives regarding strengths and concerns related to the reorganization of proximity care, including digitalization of care, with a specific emphasis on related medication safety issues.

The validity of the obtained results is supported by the study’s methodological rigor, coupled with active community engagement in all project phases. In particular, the survey co-creation process ensured that the explored issues related to proximity care implementation, digitalization, and medication care safety were genuinely relevant and of interest to patients. This approach aims to guide improvements in healthcare based on the needs and perspectives of the main stakeholders.

### 4.1 Desired participation in medication choice and level of digitalization

Among the independent variables, although most participants valued being adequately informed and involved in the medication decision process, four out of five respondents considered deciding on the best medication option a primary physician’s responsibility. Existing literature supports these findings, indicating that while patients appreciate having choices and discussing them with their physician, they prefer the physician to take the lead in final decision-making (32, 33). According to the literature, implementing telehealth consultation relies on patient engagement to actively and efficiently collect all necessary information without the support of a physical examination (34). This evidence is supported by our results showing a positive significant relationship between higher desired engagement in medication choice and the perceived usefulness of telehealth consultations.

The level of digitalization was high in our sample, and no significant differences between patients and healthy individuals were observed for any of the items. Specifically, participants reported habitually using about 4 out of 5 digital platforms and having all the necessary equipment for teleconsultations or telehealth visits. However, almost one out of ten participants reported not having an application for video calls, and nearly 40% declared they do not regularly use this digital support in their everyday lives. To realize telemedicine and telehealth’s full potential, it is a priority to focus and invest resources on developing an inclusive service that can overcome various access barriers according to the population’s specific needs (35, 36). In our study, almost one out of three participants reported not knowing how to access their EHR. A large USA study examining EHR access among nearly 30,000 patients found a similar proportion, with 35% never accessing the portal (37). Open access to HER is recognized as a valuable tool for patient engagement in chronic disease and therapy management, positively impacting patient care processes such as increased drug adherence and outcomes (38, 39).

### 4.2 Knowledge, perceived needs, and attitudes toward the reorganization of proximity care and medication safety issues

Regarding participant’s perception of outcomes of Area 1, the first notable result is that almost three-quarters of the sample experienced at least one issue related to continuity of care, either as a patient or a caregiver. These issues included difficulties in managing a chronic condition at home, the feeling of being discharged prematurely, or challenges related to continuing therapy during the transition from hospital to home setting. Intermediate care may represent a valid and effective organizational solution to face these issues. Indeed, intermediate care is specifically intended to “*facilitate patients’ transitions from illness to recovery, or to prevent their transition from home-managed chronic impairment to institution-based dependence, or to help terminally ill people be as comfortable as possible at the end of their lives*,” especially during the critical stage of care transitions (40). In this context, intermediate care pathways may play a key role in ensuring patient safety, preventing medication mismanagement, and reducing the occurrence of medication errors, particularly across care boundaries (41).

Although the term “*intermediate care*” was introduced in Italian healthcare programming documents a few years ago, and a few intermediate care facilities have already been established in Italy, only one in four respondents reported being aware of this option. Moreover, only a tiny portion of the sample (2%) stated having direct experience with it, despite the high reported rate of care discontinuation.

Engaging patients in intermediate care pathway development enables them to set realistic goals aligned with their needs, fostering improved outcomes and supporting the recovery of maximum autonomy before being transferred home (42). Disseminating sufficient information regarding the role of intermediate care facilities in patients’ journeys and their integration into the broader healthcare system is crucial for promoting their effective utilization to benefit patients’ and medication safety genuinely (43).

Concerning, more specifically, medication continuation issues, in our study, approximately one-third of participants experienced challenges related to continuing therapy during the transition from hospital to home, including medication reconciliation failure (i.e., discrepancies or mistakes that occur during the process of reviewing and documenting a patient’s medication list across transitions in care) and a lack of proper information regarding side effects and other safety concerns. Literature indicates medication reconciliation errors as a frequent and highly risky cause of severe patient harm due to, for example, incorrect dosages, duplicate therapies, or missed medications. These errors are not only harmful but also economically costly (44, 45).

Adopting a digital pharmaceutical dossier can serve as a valuable support tool for managing patient safety by addressing issues such as drug interactions, medication errors, side effects, as well as mitigating errors arising from lapses in medication reconciliation (46, 47). Similarly, pharmacists can be crucial in supporting patients with medication management (48). Specifically, pharmacists can support drug therapy management across care transitions and ensure the exchange of adequate information regarding any changes in medication therapy, as well as play a key role in medication assistance referral service (49–51). Research suggests that pharmacists’ services can have a beneficial effect on the symptoms experienced by oncology patients (52). Our data supports the value of these resources in positively impacting safe medication management. Nearly all participants agreed that the “digital pharmaceutical dossier is a valuable resource for identifying all potentially dangerous interactions or incompatibilities between medications,” and almost 95% perceived “tele-pharmacy services as useful for monitoring therapy.” However, the proportion of those who rated tele-pharmacy as ‘essential’ was much lower: 30% among patients and 50% among healthy individuals. According to the literature, acceptance and adoption of tele-pharmacy may be increased by fostering patients’ awareness of these medication-related services relevant to medication safety (53). Moreover, user manuals to guide and counsel patients on using tele-pharmacy or other educational tools are essential to promote the efficient use of all available services and improve outcomes such as medication acceptance and compliance (54, 55).

Regarding shifting therapies usually administered in hospital settings to home settings, our sample showed a generally positive attitude. Participants recognized the main advantages of transitioning to community-based care, such as simplified disease management and improved quality of life. Notably, despite this positive trend, healthy individuals were significantly more favorable toward the shift than patients. Specifically, one out of four patients disagree on the benefit of creating a familiar environment that helps tolerate medications better; similarly, the reduction of stress is recognized as a favorable element by slightly more than 50% of patients. These results suggest that patients may feel isolated in a home setting, and the development of proximity solutions might be perceived as abandonment by the healthcare system, potentially nullifying recognized advantages (i.e., avoiding prolonged hospitalizations and the related disconnection from usual social networks and living environments). Indeed, according to our study, the apprehension of feeling less supported in a home setting and concerns about the potential negative impact on the doctor-patient relationship are more pronounced among patients compared to healthy individuals. Moreover, these concerns are reported by the patients subsample with similar frequency as fears related to side effects (almost 60%). The literature analyzing similar issues is scarce. An exploratory study examining stakeholders’ perspectives on oncological home hospitalization revealed significant patient reluctance to adopt the new model (56)- Key barriers included the limited engagement of primary care in the ongoing management of cancer patients, historically centered on hospital-based treatments. Enhanced collaboration and communication between hospital and primary care were recognized as crucial prerequisites before operationalizing the model. Similarly, a review on barriers to intensive home hemodialysis identified fear of isolation and concern over inadequate professional monitoring as primary patient-related obstacles to implementing this care model (57). Similar concerns about feeling alone in managing therapy during the transition from inpatient to outpatient care were expressed by patients and family caregivers in palliative care (58). The reluctance to manage therapy at home was exacerbated by insufficient education, inadequate planning of a home medication schedule, poor coordination among home care services, and unclear communication among key stakeholders (59). These factors contributed to a sense of insecurity, uncertainty, and feelings of being overwhelmed, as well as a lack of confidence in their own or their caregivers’ ability to handle necessary treatments at home, leading them to prefer the hospital setting.

Observed results suggest the ongoing dominance of a hospital-centered culture. This mindset prioritizes hospital-based care over community-based pathways, leading to high rates of preventable and inappropriate admissions to hospitals or emergency rooms (60). Interestingly, the transition of medication administration from a hospital to an intermediate care setting was rated by our sample as less favorable than the transition toward home care, confirming that community-based care is still perceived as less supportive or less equipped by patients.

Patient engagement is essential to drive a cultural change toward embracing community-based care and effectively implementing proximity medicine and patient-centered care (PCC). Active patient participation in developing home or intermediate care programs enhances their understanding of these new organizational models, enabling early identification of critical issues and barriers and facilitating more vigorous advocacy efforts to raise awareness of community-based care and influence healthcare policies. Moreover, the literature demonstrates that community engagement in healthcare planning increases a ‘sense of ownership of the health service’ (17). This result may increase trust in community-based pathways and serve as leverage to overcome the still partially dominant hospital-centric perspective.

### 4.3 Knowledge, perceived digital evolution in proximity care development and impact on medication safety

Digitalization can play a crucial role in supporting the implementation of new proximity models and enhancing the feasibility of community-based care pathways (61). However, our study has identified several challenges that need to be adequately addressed, as shown by the results of Area 2.

Regarding implementing the already mentioned digital pharmaceutical dossier, approximately one out of five participants was concerned that it may reduce patient engagement in therapy management. According to the literature, more accessible access to clinical health data may increase patients’ perception of control (62, 63). However, several barriers may limit their usability, such as limited digital literacy and difficulties with patient portal interfaces (63). To ensure that digital patient portals are genuinely available and usable by patients, it is essential not only to promote the widespread acquisition of basic digital skills but also to ensure that the portal interfaces are intuitive and co-designed with patients (64).

Considering the preference for digital healthcare over traditional healthcare, our sample generally exhibited a positive attitude. However, as observed with the shift of medication administration from hospital to home settings, a primary concern was the need for more support. Specifically, approximately 1 out of 4 patients do not agree that digitalization enhances their sense of security and control over therapy, about 1 out of 3 express concerns about its potential to restrict self-management capabilities, and nearly half of the sample fear feeling excessively responsible for managing their illness and medications. Furthermore, despite the high level of digitalization observed among our sample, concerns regarding the creation of access disparities were noted by nearly 80% of the respondents.

To effectively address these concerns, it is crucial to promote health literacy and digital literacy while preserving or strengthening the humanization of care principles to mitigate negative perceptions and potential feelings of disconnection and detachment associated with digital healthcare (65). Indeed, common concerns and primary challenges related to health information technology often arise not from the technology itself but from how it is designed and implemented in healthcare settings (66). Similar fears can explain the significantly lower proportion of patients who perceived telehealth as “essential” compared to healthy individuals. The literature supports this result. According to a survey of over 2000 Americans, patients are inclined to use video visits but prefer in-person care (67).

Finally, the benefits of digital monitoring are well documented in the literature (68, 69). However, in our study, only 30% of patients considered this approach superior and more personalized compared to in-person monitoring, with a similar percentage considering it inferior and less attentive. A research field exploring stakeholders’ perceptions and potential barriers to digital monitoring implementation in different settings and populations is emerging. For example, a recent systematic review of the benefits and challenges of digital monitoring highlights that increased patient anxiety may represent an essential obstacle to its effective implementation (70). Patient engagement in research aimed at implementing digital monitoring in the patient pathway is critical to educate patients and healthcare providers about their changing roles, anticipate and address emerging issues promptly, and effectively guide the implementation process according to patients’ primary needs and concerns (71).

### 4.4 Variables affecting perceptions of proximity care and digitalization

Level of education and perception of health status were found to have a more extensive impact on outcomes in Area 1 and Area 2.

People with an academic degree seem more aware of and open to change brought about by implementing proximity care. A possible explanation of their higher propensity toward shifting medication administration from a hospital to a community setting may be related to a higher trust in their self-management abilities and fewer concerns about leaving the protected hospital environment for medication administration. Moreover, evidence shows that health literacy is related to higher trust in healthcare systems (72); this, in turn, may lead to more openness to change about proximity care implementation.

People with academic degrees were also more inclined toward healthcare digitalization, a result supported by existing literature (73). Studies show that individuals with higher levels of education also possess higher health and digital literacy (74, 75). This increased literacy may reduce their fear of not understanding important health information when using digital tools instead of relying on a more direct relationship with healthcare providers.

Significantly, health status perception had a greater impact on participants’ views than the mere presence of a disease. According to the literature, overall well-being is associated with higher self-management abilities and more productive interactions with healthcare providers (76); this relationship may explain the observed positive correlation between higher perceived health and a more favorable attitude toward shifting therapy to community-based settings and embracing digitalized care. Moreover, evidence shows that higher well-being is linked to greater resilience and capacity to adapt to new situations, which may foster a more positive attitude toward reorganizing proximity care, perceived by people with higher health status as an opportunity rather than a concern (77).

Regarding the type of pathology, people suffering from an oncological disease were generally less inclined toward the digitalization of care, including therapy management, compared to healthy individuals and patients with non-oncological diseases. Suffering from a life-threatening disease may create a higher need for empathy and closeness, which is more easily conveyed through in-person consultations. For example, a study exploring oncological patients’ experience using telehealth therapy sessions compared to in-person sessions evidenced a few limitations, including “*less opportunity for personalization*” (78). On the other hand, being either healthy or affected by less threatening chronic conditions may lead patients to prefer a more straightforward, albeit more ‘impersonal,’ way to monitor their therapy or communicate with HCPs.

Finally, in general, healthy individuals showed a trend toward a greater openness regarding the development of proximity care compared to those affected by a disease, as visually demonstrated in Figure 3. This finding may partly be explained by the fact that patients, due to their more frequent interactions with healthcare services, are more exposed to eventual inefficiencies and perceived limitations of the healthcare system. However, these results should be interpreted with some caution, particularly considering the smaller sample size and potential representativeness of healthy individuals in the survey.

### 4.5 Strengths and limitations

The study’s main limitation concerns the potential selection bias related to the data collection method. The use of an electronic questionnaire and the mediation by patient associations may have favored the selection of more aware and experienced participants who are thus more favorable toward proximity care and digitalization-related issues. Similarly, not all regions were equally represented, and the impact of varying local (regional) healthcare service organizations dedicated to proximity care, as perceived by the participants, could not be analyzed in detail. Moreover, due to selection bias, not all diseases were equally represented, partly limiting the power of subgroup analyses (e.g., the distinction between oncological and non-oncological patients or between healthy individuals and those affected by a disease). However, the study sample, adequately sized based on the power calculation, appears sufficiently heterogeneous across various sociodemographic and clinical characteristics such as age, education level, and duration of illness, increasing the generalizability of the results. Additionally, approximately 40% of the sample was not affiliated with any patient association, and according to the regression model, this characteristic had only a marginal impact on the results. Furthermore, as expected, the sample mainly comprised individuals affected by significant health conditions (high level complexity care), considering their comorbidities and the number of medications taken. These individuals also represent key stakeholders in proximity care reorganization efforts. Finally, it is notable that the core strength of the paper, as highlighted by the PSC, lies in its attempt to synthesize the perspectives of the general population, with a specific focus on patients with chronic conditions, regardless of their underlying pathology. This cross-pathology approach was considered innovative by the PAs involved in the study, as they are not typically accustomed to collaborating transversally across different disease areas. The opportunity to compare diverse viewpoints was recognized by the PSC as a key strength of the ProSafe study.

Despite the addressed limitations in generalizing the results, evidence regarding the perspectives of patients, citizens, and caregivers on proximity medicine and drug safety is, at present, limited. The findings from the ProSafe study offer valuable insights into this crucial topic and represent a significant contribution to advancing truly patient-centered care.

## 5 Conclusion

From an academic perspective, this study contributes to the growing body of knowledge on patient-centered care by providing empirical insights into the barriers and facilitators of proximity care adoption, emphasizing the importance of co-creation methodologies in healthcare research.

These findings also offer practical implications for managers and policymakers, highlighting the challenges that must be addressed to support the transition toward proximity care. According to our results, implementing proximity care and healthcare digitalization in the context of safe medication management based on the expectations and needs of patients and citizens requires raising awareness of the role of intermediate care and developing new competencies and resources to address the concerns, fears, and limitations identified in our exploratory study.

On a societal level, this study underscores the importance of integrating patients’ voices in healthcare transformation processes, reinforcing the need for healthcare models that empower communities. Integrating patients’ perspectives as a guide for effectively planning and implementing proximity care pathways is essential to bring health, disease management, and medication management closer to the patient and their living environment. Accordingly, the ProSafe project envisions the next development phase to identify how to effectively address the critical issues identified in this study through the partnership with Patient Associations, relevant Institutions, Academics, and Pharma Companies. This collaborative approach ensures that the community becomes co-creators in the implementation of proximity care fostering health equity and patient autonomy.

Additionally, a further development of the ProSafe study will explore the perspectives of other essential stakeholders, such as healthcare providers. Comparing the perspectives of key stakeholders will provide a more comprehensive view of the main issues related to this critical reorganization of care, ensuring that healthcare providers and patients can proceed in partnership throughout the entire process.

## Data Availability

The original contributions presented in the study are included in the article/Supplementary material, further inquiries can be directed to the corresponding author/s.
