# Supplementary material for: Proximity care pathways and digitalization: opportunities and concerns for medication safety management—Insights from the ProSafe study on community perspectives
Source: Front Public Health. 2025 Feb 20;13:1486814. doi: 10.3389/fpubh.2025.1486814 (PMC11882513; doi:10.3389/fpubh.2025.1486814)
Supplement: Supplementary file 1 [file Data_Sheet_1.pdf]

Supplementary Material

1 Supplementary Figures and Tables

1.1 Supplementary Tables

Table 1S. Distribution of main results by the subgroups of patients and healthy individuals

	**Mean (SD) or % Patients**	**Mean (SD) or % healthy individuals**	**t-test or z-test (p-value)**
SECTION 2			
Desired participation in medication choice	3,2 (0,4)	3,1 (0,4)	1,27 (0,21)
Level of digitalization			
*Digital platforms habitually used**	3,9 (1,3)	4,0 (1,2)	0,69 (0,49)
know how to access their EHR (% yes)	70,6%	79,7%	1,63 (0,10)
*Having all the necessary equipment for a telemedicine visit***	3,8 (0,6)	3,8 (0,6)	0,75 (0,45)
SECTION 3			
Area 1. Knowledge, perceived needs, and attitudes toward the reorganization of proximity care and medication safety issues			
Knowledge of IF (% yes)	23,6%	25,3%	0,52 (0,61)
issues with continuity of care from hospital to Home/Community (% yes, at least one issue)	71,2%	65,4%	1,00 (0,32)
Attitudes toward shifting hospital therapy to the home-setting	2,8 (0,6)	3,0 (0,5)	**3,12 (<0,01)**
attitudes toward shifting hospital therapy to the IF setting	2,7 (0,7)	3,0 (0,6)	**3,78 (<0,02)**
Area 2. Digital Evolution in proximity care development and impact on medication safety			
Propensity toward the implementation of the medication dossier (% of completely favorable[§])	39,1%	44,3%	0,85 (0,40)
Perceived usefulness of telehealth visits/consultations for monitoring therapy	1,3 (0,5)	1,5 (0,4)	**3,03 (<0,01)**
Propensity toward digital medicine (vs traditional medicine)	3,0 (0,5)	3,1 (0,5)	**2,12 (<0,03)**
Propensity toward digital monitoring (vs in-person monitoring) - % of "better and more personalized	24,7%	32,9%	1,49 (0,16)

*IF=Intermediate Facilities; *Among 5 choices: emails, instant messaging services, social media, search engines, and platforms for teleconferencing or video calls (from 0 to 5); ** Having an internet connection, a PC smartphone or tablet with a photo camera, and an application for video calls; §Percentage calculated as the proportion of people who completely agree with positive items and completely disagree with negative items*

Table 2S. Distribution of results by items (among the total sample and the subsamples of patients and healthy individuals)

**Area/ variable**	**Items**	**Total**	**Patients (Mean or %)**	**healthy individuals (Mean or %)**	**Test-value* (p-value)**
Desired participation in medication choice	*Item 1. It is entirely the doctor's responsibility to choose the best medication option.* ***Mean (SD) – negative item****.*	3,11 (0,88)	3,12 (0,90)	3,08 (0,86)	0,39 (<0,69)
	Item 2. It's important to be informed about all the possible side effects of medications. ***Mean (SD)***	3,82 (0,61)	3,86 (0,54)	3,70 (0,82)	***2,08 (<0,01)***
	Items 3. Being involved in decisions about medications increases my confidence and reduces the likelihood of interruption. ***Mean (SD)***	3,69 (0,72)	3,71 (0,69)	3,63 (0,80)	0,84 (0,40)
Level of digitalization	***Digital platforms habitually used (% yes)***				
	E-mail	95,0%	95,3%	93,7%	0,58 (0,56)
	Instant messaging services (e.g., WhatsApp, Telegram, Messenger...etc.)	90,2%	89,0%	94,9%	-1,58 (0.11)
	Social media (es. Facebook, Instagram, Twitter)	60,2%	59,6%	63,3%	-0,60 (0,55)
	Search engines (e.g., Google, Bing, Edge).	83,9%	83,7%	84,8%	-0,24 (0,80)
	Platforms for video calls/video conferences (e.g., Google Meet, Zoom)	59,5%	59,1%	60,8%	-0,28 (0,78)
	know how to access their EHR (%)				
	Yes	72,4%	70,6%	79,8%	2,66 (0,26)
	No, but I know what it is	17,3%	18,4%	12,7%	
	No, and I don't know what it is	10,3%	11,0%	7,6%	
	Having all the necessary equipment for a telemedicine visit (% yes)				
	Internet connection,	98,3%	98,5%	97,5%	0,65 (0,51)
	Photo-camera,	94,7%	94,3%	96,2%	-0,67 (0,50)
	PC smartphone or tablet	98,6%	98,8%	97,5%	0,89 (0,37)
	Application for video calls	89,4%	88,9%	92,4%	-0,92 (0,82)

Area/ variable	Items	Total	Patients (Mean or %)	healthy individuals (Mean or %)	Test-value* (p-value)
Area 1. Knowledge, perceived needs, and attitudes toward the reorganization of proximity care and medication safety issues	***Knowledge of IF (Have you ever heard about intermediate facilities?) (%)***				
	Yes, and I know what they are	23,1%	22,6%	25,3%	0,98 (0,61)
	Yes, but I haven't quite understood what they are.	25,1%	24,4%	27,9%	
	No	51,8%	53,0%	46,8%	
	issues with continuity of care from hospital to Home/Community setting (% yes)				
	Item 1. Sudden worsening of a chronic disease that is difficult to manage at home but not 'serious enough' to warrant hospitalization	50,6%	56,1%	27,8%	***4,52 (<0,01)***
	Item 2. Feel discharged from the hospital too soon	42,4%	41,4%	48,1%	-1,09 (0,28)
	Item 3. Being hospitalized and then encountering difficulties in continuing pharmacological treatment at home	31,8%	30,7%	35,9%	0,80 (0,37)
	Attitudes toward shifting hospital therapy to home-setting - Mean score (SD)				
	It would improve the quality of life	3,27 (0,79)	3,23 (0,81)	3,43 (0,71)	***-2,03 (<0,05)***
	It would simplify disease management	3,25 (0,81)	3,21 (0,84)	4,41 (0,65)	***-1,96 (=0,05)***
	It would create a familiar environment that would help better tolerate the medication	3,09 (0,90)	3,00 (0,93)	3,42 (0,61)	***-3,71 (<0,01)***
	It would decrease the level of stress	2,68 (0,99)	2,58 (1,00)	3,09 (0,82)	***-4,20 (<0,01)***
	I would feel less supported in the care process ***(negative item)***	2,65 (0,97)	2,68 (0,98)	2,48 (0,90)	1,69 (0,09)
	I would be more fearful of the side effects ***(negative item)***	2,75 (0,98)	2,73 (1,01)	2,84 (0,82)	-0,88 (0,38)
	It would negatively affect the doctor-patient relationship ***(negative item)***	2,34 (1,00)	2,38 (1,03)	2,15 (0,86)	1,82 (0,07)
	In general, it is a useful change	3,15 (0,79)	3,11 (0,80)	3,33 (0,71)	***-2,20 (<0,01)***
	Attitudes toward shifting hospital therapy to IF setting - Mean score (SD)				
	It would improve the quality of life	2,96 (0,88)	2,90 (0,88)	3,21 (0,86)	***-2,74 (<0,01)***
	It would simplify disease management	3,00 (0,91)	2,93 (0,92)	3,31 (0,79)	***-3,34 (<0,01)***

Area/ variable	Items	Total	Patients (Mean or %)	healthy individuals (Mean or %)	Test-value* (p-value)
	It would decrease the level of stress	2,54 (1,03)	2,45 (1,04)	2,95 (0,87)	***-3,93 (<0,01)***
	I would feel less supported in the care process ***(negative item)***	2,49 (1,08)	2,51 (1,08)	2,38 (1,08)	0,99 (0,32)
	I would be more fearful of the side effects ***(negative item)***	2,36 (1,01)	2,40 (1,00)	2,19 (1,02)	1,64 (0,10)
	It would negatively affect the doctor-patient relationship ***(negative item)***	2,33 (1,06)	2,40 (1,05)	2,05 (1,02)	***2,61 (<0,01)***
	In general, it is a useful change	2,99 (0,88)	2,92 (0,89)	3,27 (0,82)	***-3,14 (<0,01)***
Digital Evolution in proximity care development and impact on medication safety	***Propensity toward the implementation of the medication dossier (How much do you agree with the following statements?) – Mean (SD)***				
	Item 2. It is a valuable resource to identify all potentially dangerous interactions or incompatibilities between medications	3,58 (0,64)	3,59 (0,639	3,57 (0,67)	0,23 (0,82)
	Item 1. It is an initiative that risks reducing patient involvement in therapy management. ***(negative item)***	1,79 (1,69)	1,81 (0,97)	1,71 (0,96)	0,81 (0,42)
	Perceived usefulness of telehealth visits/consultations for monitoring therapy – Mean (SD)				
	Teleconsultation with a general practitioner (GP) or specialist.	1,34 (0,58)	1,31 (0,57)	1,48 (0,60)	***-2,44 (<0,05)***
	Teleassistance with nurses or other local healthcare providers	1,28 (0,59)	1,24 (0,59)	*1,46 (0,57)*	***-2,96 (<0,01)***
	*Tele-pharmacy service**	1,29 (0,60)	1,25 (0,60)	*1,43 (0,55)*	***-2,41 (<0,05)***
	Propensity towards digital medicine (vs traditional medicine) – Mean (SD)				
	It allows for limiting hospital visits	3,36 (0,82)	3,34 (0,83)	3,47 (0,73)	-1,27 (0,21)
	It instills a greater sense of security by ensuring more control over medication therapy	2,89 (0,83)	2,86 (0,84)	3,01 (0,78)	-1,51 (0,13)
	It helps manage medication therapy more independently	3,13 (0,79)	3,08 (0,80)	3,33 (0,73)	***-2,49 (<0,05)***
	It saves times	3,43 (0,74)	3,39 (0,76)	3,59 (0,63)	***-2,28 (<0,05)***
	It allows for easy access to all health and medication information	3,36 (0,70)	3,34 (0,71)	3,46 (0,66)	-1,30 (0,19)

Area/ variable	Items	Total	Patients (Mean or %)	healthy individuals (Mean or %)	Test-value* (p-value)
	It raises concerns regarding privacy protection ***(negative item)***	2,01 (1,06)	2,03 (1,05)	1,92 (1,08)	0,78 (0,44)
	It places excessive responsibility on managing one's own health or disease ***(negative item)***	2,26 (0,99)	2,28 (1,01)	2,16 (0,90)	0,96 (0,34)
	It risks leading to a constant need for help with health-related activities, limiting autonomy ***(negative item)***	1,97 (0,97)	1,99 (0,97)	1,92 (1,00)	0,50 (0,62)
	It risks creating disparities by targeting only those who can access care because they have the necessary tools ***(negative item)***	3,04 (0,88)	3,06 (0,88)	2,96 (0,87)	0,91 (0,36)
	Propensity toward digital monitoring (compared to in-person monitoring) (%)				
	"better and more personalized."	26,2%	24,6%	32,9%	3,86 (0,28)
	"I would receive the same assistance."	31,3%	31,8%	29,1%	
	"worse and less attentive"	25,2%	25,0%	26,6%	
	"Not sure"	17,3%	18,7%	11,4%	

** t-test o z-score or Chi2 score according to variable type*

Table 3S. Results of the Conditional Mixed Process (CMP) model conducted for outcomes in Area 1 ("Knowledge, perceived needs and attitudes toward the reorganization of proximity care and medication safety issues").

Outcomes	Variables	Coeff	95% CI
**Knowledge of IF**	***Sex***		
	M	-0,28	-0,60; -0,43
	F	0	
	Age class		
	18-29	0	
	30-49	0,54	-0,27; 1,36
	50-69	0,91	0.12; 1,70
	≥70	1,32	0,44; 2,20
	Academic degree		
	ESa/JHSb Diploma	-7,2	-1,38;-0,55
	High School Diploma	-0,2	-0,34; 0,31
	University/ PGc Degree	0	
	Health Status Self Perception	0,2	0,13; 0,41
	know how to access their EHR	0,58	0,20; 0,96
	Constant (Intercept)	-2,4	-3,47;-1,35
**Issues with continuity of care from hospital to Home/Community**	***Sex***		
	M	-0,4	-0,65;-0,51
	F	0	
	Age class		
	18-29	0	
	30-49	-0,24	-0,88; 0,41
	50-69	-0,60	-1,21; 0,18
	≥70	-0,80	-1,53;-0,76
	Member of a Patients' Association	0,34	0,35; 0,64
	Health Status Self Perception	-0,35	-0,55;-0,16
	Desired participation in medication choice	0,42	0,07; 0,77
	Constant (Intercept)	0,57	-0,85; 1,99
**Attitudes toward shifting hospital therapy to the home-setting**	***Academic degree***		
	ESa/JHSb Diploma	-0,21	-0,39;-0,04
	High School Diploma	-0,09	-0,19; 0,00
	University/ PGc Degree	0	
	Health Status Self Perception	0,10	0,03; 0,17
	Constant (Intercept)	2,65	2,45; 2,85
**Attitudes toward shifting hospital therapy to the IF setting**	***Health Status Self Perception***	0,14	0,55; 0,23
	Time since diagnosis		
	Healthy	0	
	< 1 year	-0,46	-0,79;-0,13
	1-5 years	-0,27	-0,50;-0,04
	6-10 years	-0,20	-0,45; 0,64
	>10 years	-0,39	-0,63;-0,15
	ADR/side effects		
	Healthy	0	
	Medication at home	0,35	0,15; 0,55
	Medication in a hospital setting	0,16	-0,04; 0,36
	Constant (Intercept)	2,49	2,23; 2,77

Table 4S. Results of the Conditional Mixed Process (CMP) model conducted for outcomes in Area 2 ("Digital Evolution in proximity care development and impact on medication safety").

Outcomes	Variables	Coeff	95% CI
**Propensity toward the implementation of the digital pharmaceutical dossier**	***Academic degree***		
	ESa/JHSb Diploma	-0,34	-0,80; 0,12
	High School Diploma	-0,32	-0,57;-0,06
	University/ PGc Degree	0	
	Health Status Self Perception	0,17	0,02; 0,33
	Constant (Intercept)	-0,51	-0,95;-0,06
**Perceived usefulness of telehealth visits/consultations for monitoring therapy**	***Sex***		
	M	-0,15	-0,24;-0,51
	F	0	
	Academic degree		
	ESa/JHSb Diploma	-0,08	-0,27; 0,11
	High School Diploma	-0,10	-0,20;-0,00
	University/ PGc Degree	0	
	Role of caregiver	0,16	0,58; 0,26
	Main pathology		
	Healthy	0	
	*Non-oncological	-0,13	-0,25;-0,01
	Oncological	-0,21	-0,35;-0,06
	Desired participation in medication choice	0,13	0,02; 0,24
	Having all the necessary equipment for a telemedicine visit	0,14	0,06; 0,22
	Constant (Intercept)	0,54	0,51; 1,03
**Propensity toward digital medicine (vs traditional medicine)**	***Age class***		
	18-29	0	
	30-49	-0,02	-0,19; 0,14
	50-69	-0,07	-0,23; 0,09
	≥70	-0,23	-0,44;-0,03
	Academic degree		
	ESa/JHSb Diploma	-0,02	-0,19; 0,16
	High School Diploma	-0,14	-0,24;-0,05
	University/ PGc Degree	0	
	Health Status Self Perception	0,07	0,01; 0,12
	Main pathology		
	Healthy	0	
	*Non-oncological	-0,00	-0,11; 0,11
	Oncological	-0,14	-0,28;-0,00
	Having all the necessary equipment for a telemedicine visit	0,19	0,12; 0,27
	Constant (Intercept)	2,25	1,87; 2,64
**Propensity toward digital monitoring (vs in-person monitoring) -**	***Role of caregiver***	0,29	0,36; 0,55
	Having all the necessary equipment for a telemedicine visit	0,26	0,02; 0,51
	Constant (Intercept)	-1,72	-2,68;-0,77

*Including Hereditary/congenital disease

1.2 Supplementary Figures

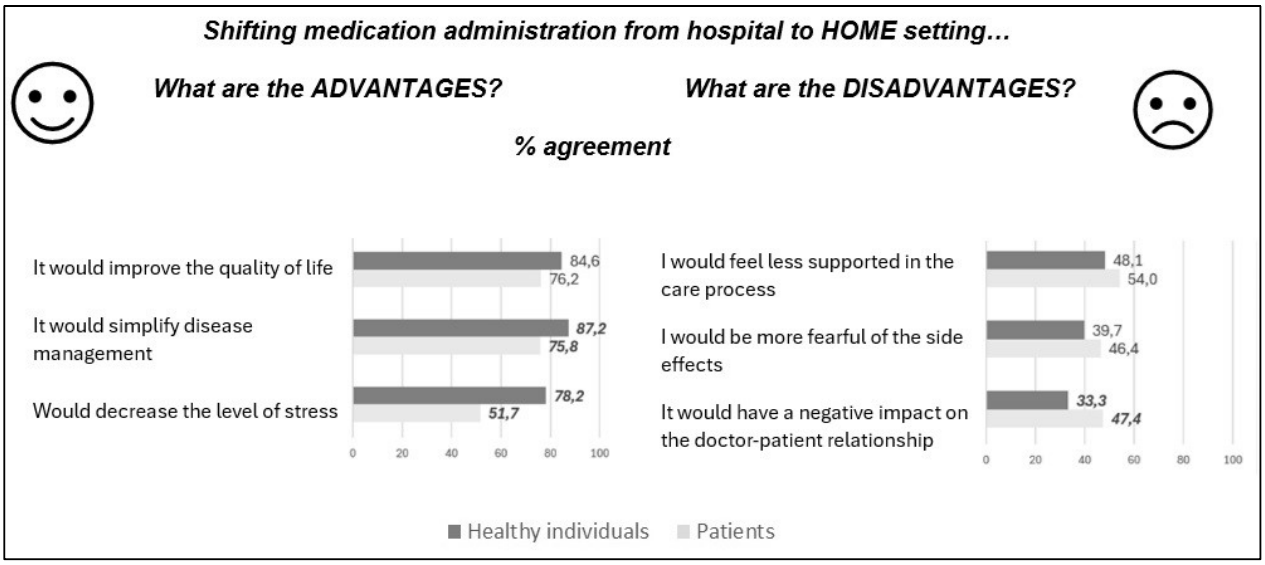

Supplementary Figure 1. Distributions of answers (% of agreement) regarding advantages and disadvantages of shifting medication administration from hospital to IF setting by subpopulations of patients and healthy individuals. Significant differences between patients and healthy individuals are highlighted in bold.

1.3 Diagnostic analysis of the CMP models

1.3.1 Area 1

Supplementary Figure 2. Q plot of the residuals

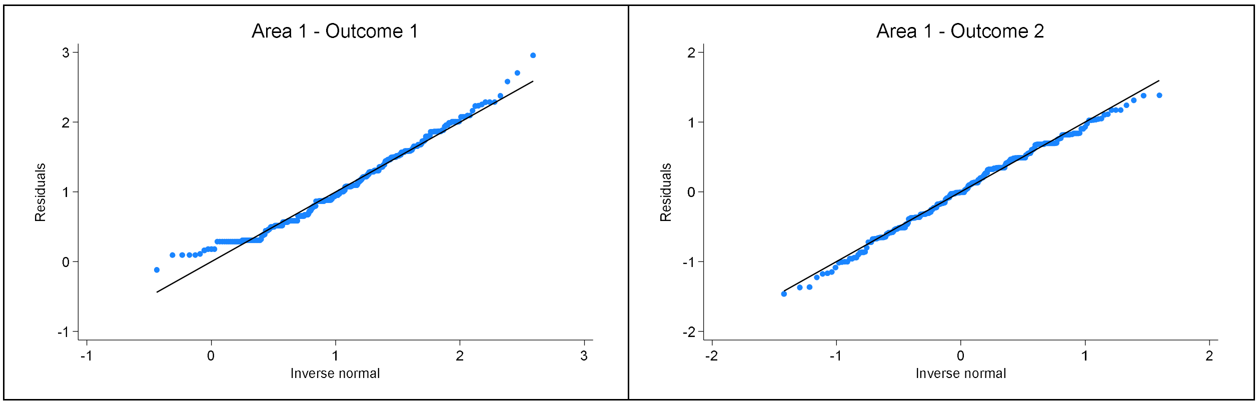

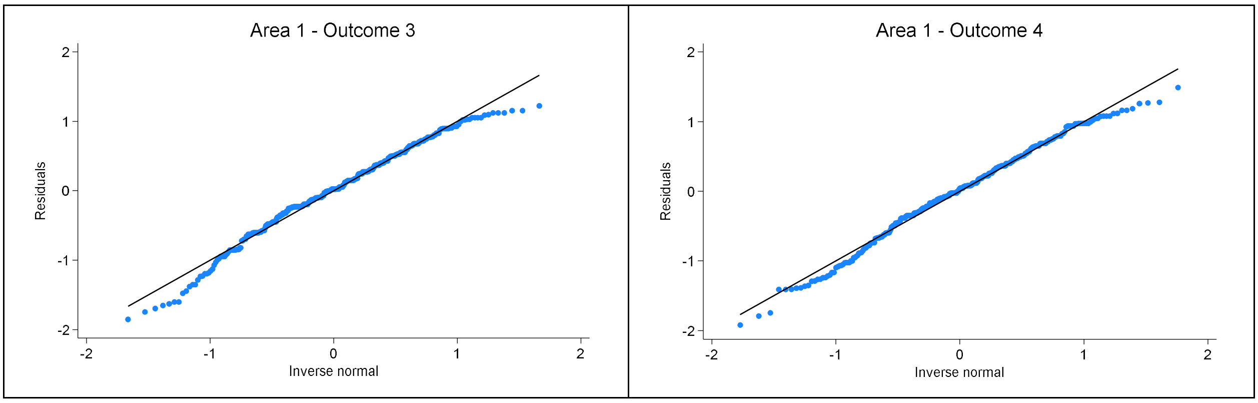

Outcome 1: Knowledge of IF; Outcome2: Issues with continuity of care from hospital to Home/Community; Outcome3: Attitudes toward shifting hospital therapy to the home-setting; Outcome4: Attitudes toward shifting hospital therapy to the IF setting

Table 5S. Correlation between residuals of the 4 outcomes variables

	Outcome 1	Outcome 2	Outcome 3
Outcome 2	-0,15*		
Outcome 3	-0.03	0.11*	
Outcome 4	-0.07	0.06	0.62*

*significant correlations (p<0.05)

1.3.2 Area 2

Supplementary Figure 3. Q plot of the residuals

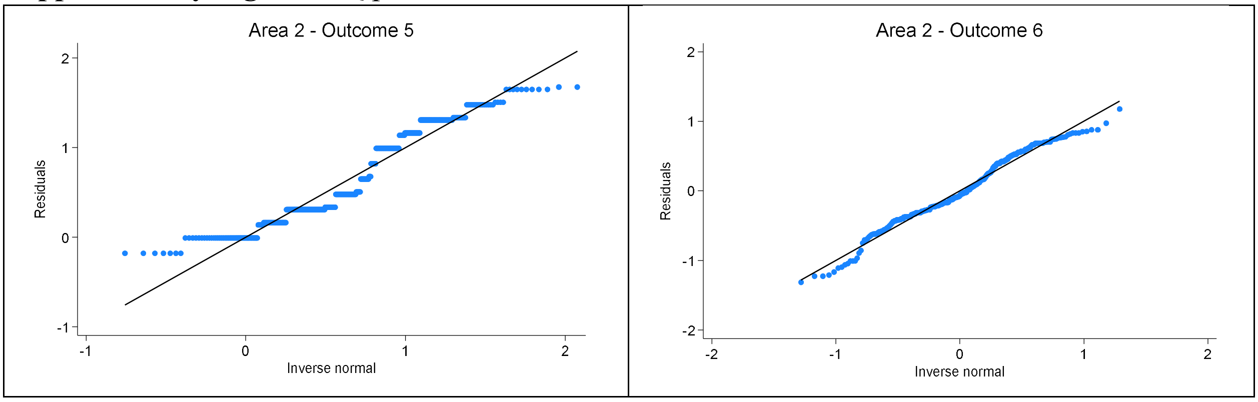

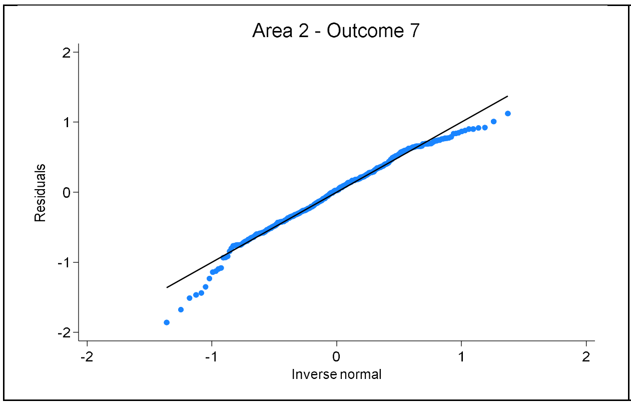

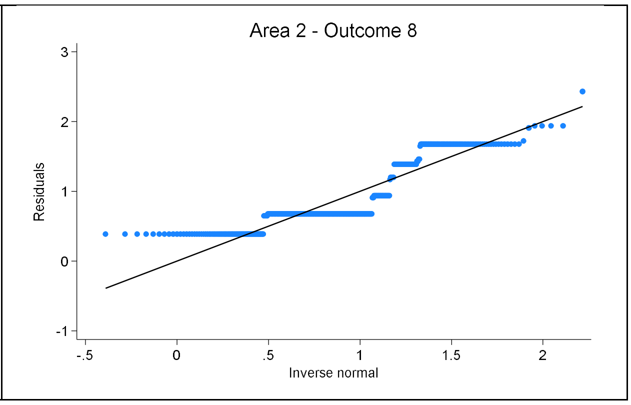

Outcome5: Propensity toward the implementation of the digital pharmaceutical dossier; Outcome6: Perceived usefulness of telehealth visits/consultations for monitoring therapy; Outcome7: Propensity toward digital medicine (vs traditional medicine); Outcome8: Propensity toward digital monitoring (vs in-person monitoring).

Table 6S. Correlation between residuals of the 4 outcomes variables

	Outcome 1	Outcome 2	Outcome 3
Outcome 2	0,13*		
Outcome 3	0.33*	0.33*	
Outcome 4	0.13*	0.15*	0.38*

*significant correlations (p<0.05)
